# Voltage-gated potassium channels as a potential therapeutic target for the treatment of neurological and psychiatric disorders

**DOI:** 10.3389/fncel.2024.1449151

**Published:** 2024-10-01

**Authors:** Isabel E. Faulkner, Rachael Z. Pajak, Michael K. Harte, Jocelyn D. Glazier, Reinmar Hager

**Affiliations:** ^1^Division of Evolution, Infection and Genomics, School of Biological Sciences, Faculty of Biology, Medicine and Health, The University of Manchester, Manchester, United Kingdom; ^2^Division of Pharmacy and Optometry, School of Health Sciences, Faculty of Biology, Medicine and Health, The University of Manchester, Manchester, United Kingdom

**Keywords:** Kv3 channels, schizophrenia, autism, epilepsy, ataxia, cognition, neurodevelopment

## Abstract

Voltage-gated potassium channels are a widely distributed subgroup of potassium channels responsible for the efflux of potassium in the repolarisation of the cell membrane, and hence contribute to the latency and propagation of action potentials. As they are causal to synaptic transmission, alterations to the structure of these channels can lead to a variety of neurological and psychiatric diseases. The Kv3 subfamily of voltage-gated potassium channels are found on many neurons in the brain, including inhibitory interneurons where they contribute to fast-frequency firing. Changes to the firing ability of these interneurons can lead to an imbalance of inhibitory and excitatory neurotransmission. To date, we have little understanding of the mechanism by which excitatory and inhibitory inputs become imbalanced. This imbalance is associated with cognitive deficits seen across neurological and neuropsychiatric disorders, which are currently difficult to treat. In this review, we collate evidence supporting the hypothesis that voltage-gated potassium channels, specifically the Kv3 subfamily, are central to many neurological and psychiatric disorders, and may thus be considered as an effective drug target. The collective evidence provided by the studies reviewed here demonstrates that Kv3 channels may be amenable to novel treatments that modulate the activity of these channels, with the prospect of improved patient outcome.

## 1 Introduction

Current treatments of psychiatric and neurological diseases with a cognitive deficit component such as schizophrenia (SCZ), autism spectrum disorder (ASD) and some forms of epilepsy, rely on symptom control, for example, antipsychotics for SCZ (Gomes and Grace, 2021). These treatments tend to alleviate acute symptoms such as psychosis for SCZ, but do not work for other symptom domains such as cognitive deficits. Thus, despite the large number of over 20 first and second generation antipsychotics available, none are effective in treating the full range of symptoms and some, such as cognitive deficits, remain difficult to treat. Our lack of understanding the mechanisms underlying in particular cognitive deficits contributes to this. Despite many drugs being developed to treat the cognitive deficits seen in SCZ, many other neurological and psychiatric diseases could also benefit from these treatments. In this review, we consider the central role that voltage-gated potassium channels play in the biological mechanisms and aetiology of many psychiatric and neurological disorders. Furthermore, we draw attention to investigations that examine modulation of voltage-gated potassium channel activity as a potential therapeutic target. Hence, accumulating evidence suggests that potassium channels should be at the centre of new research investigations, and further pre-clinical studies should focus on their potential as an efficacious drug target (Deakin et al., 2019).

## 2 Voltage-gated potassium channels

Transmembrane ion channels play a fundamental role in cell physiology through the transport of ions (e.g., potassium, calcium, sodium) in or out of the cell. In particular, voltage-gated potassium (Kv) channels that open or close in response to changes in the electrical membrane potential play a critical role in excitable cells, especially in neurons, for the establishment of membrane potential and the repolarisation of the cell membrane following an action potential, ensuring the return of the membrane to the resting state (Clatot et al., 2023). The human genome contains over 70 different genes associated with Kv channels (Gunthorpe, 2022). Kv channel genes are divided into 12 families, Kv1 to Kv12 (Liang et al., 2024). Members of the Kv3 subfamily are unique in having a high activation potential (>−10 mV), contributing to high-frequency firing (>100 Hz) and rapid deactivation, which are essential properties for the function of the cells in which they are expressed (Kaczmarek and Zhang, 2017; Chen Y.-T. et al., 2023). Kv3 channels are expressed in neurons that need to fire at high frequencies, such as cerebellar Purkinje cells, hippocampal and prefrontal parvalbumin-expressing interneurons (PVI), basal ganglia nuclei, reticular thalamic nucleus and principal neurons of several auditory brainstem nuclei (Zhang et al., 2021; Clatot et al., 2023). The ability of neurons to fire rapidly plays a crucial role in mechanisms underpinning learning and memory in the hippocampus through cortical inhibition, motor co-ordination in the cerebellum and sensory processing in the auditory nuclei (Chi et al., 2022).

**Figure 1:**
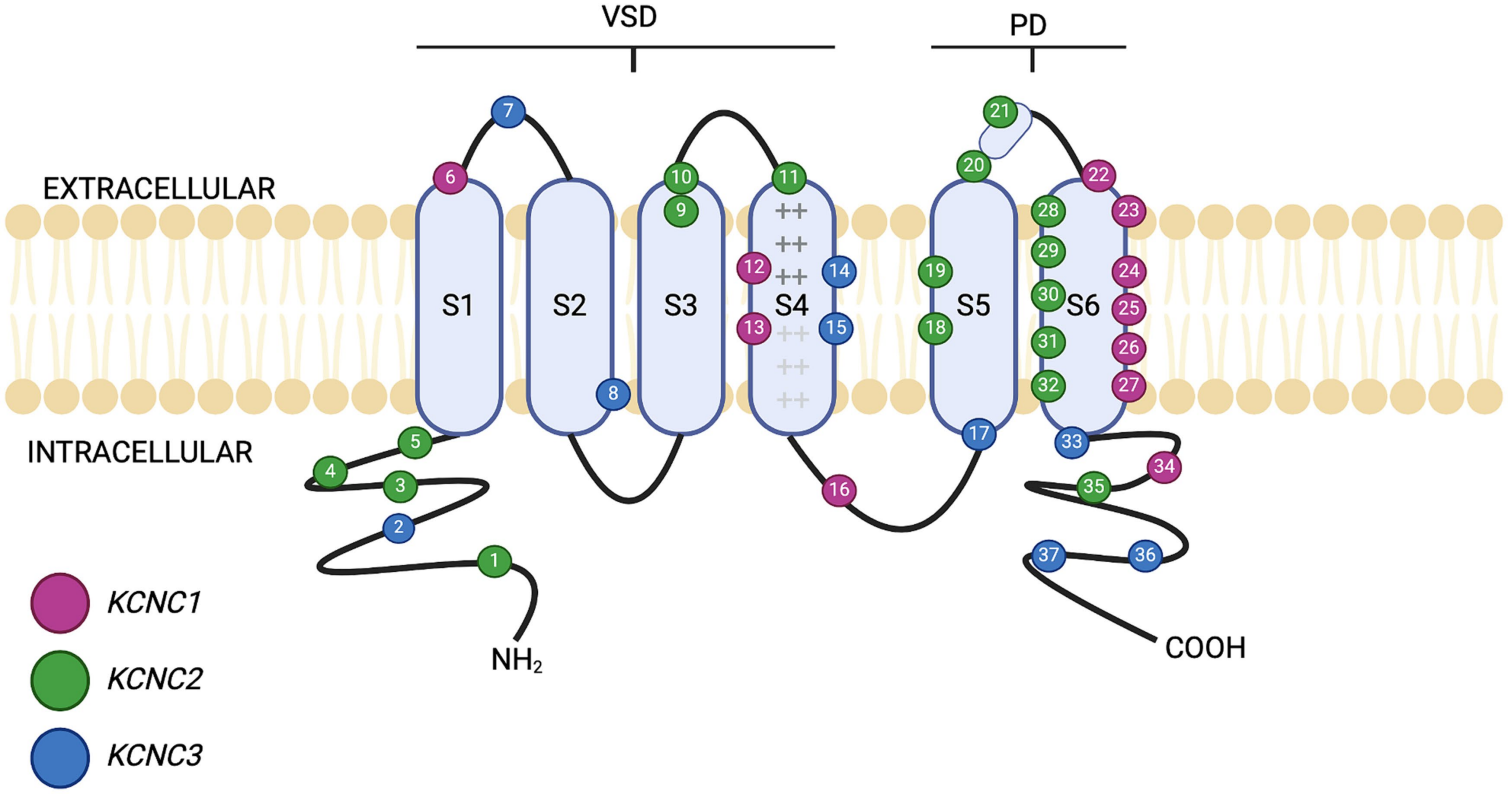
Schematic of Kv3 channel structure. The six transmembrane domains (S1–S6) are shown, along with the intracellular (cytosolic) distribution of the amino (–NH2) and carboxyl (–COOH) termini. The presence of basic amino acids arginine and lysine within segment S4 are represented by “+” notation. S1–S4 form the voltage sensing domain (VSD). Between S5 and S6 lies the pore domain (PD). The location of different mutations on Kv3.1, Kv3.2 and Kv3.3 is shown by numbered positions, colour-coded for each individual gene. Further detail of the clinical presentation of each mutation is given in Tables 1–3. Figure created using Biorender.com.

There are four subfamilies of Kv3 channels, Kv3.1, Kv3.2, Kv3.3 and Kv3.4, which are encoded by the *KCNC1*, *KCNC2*, *KCNC3* and *KCNC4* genes, respectively (Gunthorpe, 2022). The functional Kv3 channel consists of four alpha subunits which form a pore permeable to K^+^ ions (Grizel et al., 2014; Irie et al., 2014). Kv3 channels have either a homotetrameric (all the same Kva subunits), or a heterotetrameric (combination of Kv3 subunits), structure. All the Kv3 subunits contain a N-terminal cytoplasmic T1 domain, followed by six transmembrane segments, S1–S6 (Chi et al., 2022), as shown in Figure 1. S1–S4 make up the voltage sensing domain (VSD), with S4 being the primary voltage sensor (Liang et al., 2024). S4 has six positively charged arginine and lysine residues (represented by the “+” symbol within S4 in Figure 1), found at every third position within the amino acid sequence (Li et al., 2021). The positively charged arginine residues detect changes to membrane potential, resulting in the re-orientation allowing the channel to open (Aggarwal and MacKinnon, 1996). Between S4 and S5 is a linker domain that connects to the S6 pore-forming unit, with S5 and S6 forming the pore domain (Mukherjee et al., 2022). The domains are arranged so that S5 and S6 are in the centre of the channel and are peripherally surrounded by S1 to S4 (Grizel et al., 2014). This forms a selective filter that allows the exclusive transfer of K^+^ in response to changes in membrane potential (Tombola et al., 2005). The unique structure and properties of these transmembrane segments allow Kv3 channels to sustain high-frequency neuronal firing by allowing rapid repolarisation of action potentials. This rapid repolarisation is vital for neurons that require fast signalling and precise timing, such as those involved in auditory processing and fast-spiking interneurons. Therefore, any alterations in Kv3 channel structure through genetic mutations can lead to phenotypic changes affecting neuronal excitability and the overall function of neural circuits, potentially resulting in neurological and psychiatric disorders.

### 2.1 Kv3 potassium channel variants

Mutations can affect Kv3 channel function to fire at high frequencies, altering neural physiology and brain circuitry. Mutations in *KCNC1*, *KCNC2* and *KCNC3* have been implicated in several neurological and psychiatric disorders such as epilepsy, ataxia, and neurodevelopmental disorders (NDD) such as ASD and SCZ (Tables 1–3). Currently, no known clinical disorders have been implicated with mutations in *KCNC4*. Known mutations associated with different transmembrane segment domains of Kv3 channels are shown in Figure 1.

**Table 1:** *KCNC1* channel variants and associated clinical presentation.

No. on Figure 1	Protein variant	Coding DNA change	Position in Kv3.1 protein	Clinical presentation	References
6	p.Cys208Tyr	c.623G>A	S1	DE	Park et al. (2019)
12	p.Arg317Ser	c.949C>A	S4	MEAK	Li et al. (2021)
12	p.Arg317His	c.950G>A	S4	DE	Cameron et al. (2019)
13	p.Arg320His	c.959G>A	S4	MEAK PME	Muona et al. (2015), Nascimento and Andrade (2016), Oliver et al. (2017), Kim et al. (2018), and Barot et al. (2020)
16	p.Arg339*	c.1015C>T	S4–S5	DE	Poirier et al. (2017)
22	p.Thr399Met	c.1196C>T	S5–S6	DE	Park et al. (2019)
23	p.Ala421Val	c.1262C>T	S6	DEE	Cameron et al. (2019) and Park et al. (2019)
24	p.Val425Met	c.1273G>A	S6	DEE	Ambrosino et al. (2023)
25	p.Met430Ile	c.1290G>A	S6	Developmental delay	Clatot et al. (2023)
26	p.Val432Met	c.1294G>A	S6	Developmental delay + ID	Clatot et al. (2023)
27	p.Val434Leu	c.1300G>C	S6	Developmental Delay + ID	Clatot et al. (2023)
34	p.Gln492X	c.1474C>T	C terminal	DE	Cameron et al. (2019)

DD, developmental encephalopathy; DEE, developmental encephalopathy and epilepsy; ID, intellectual disability; MEAK, myoclonus epilepsy and ataxia due to potassium channel mutation; PME, progressive myoclonic epilepsy; *, undetermined amino acid change.

**Table 2:** *KCNC2* channel variants and associated clinical presentation.

No. on Figure 1	Protein variant	Coding DNA change	Position in Kv3.2 protein	Clinical presentation	References
1	p.Cys125Tyr	c.374G>A	T1 domain	DEE	Clatot et al. (2024)
1	p.Cys125Trp	c.375C>G	T1 domain	EOAE	Schwarz et al. (2022)
3	p.Glu135Gly	c.404A>G	T1 domain	DEE	Schwarz et al. (2022)
4	p.Asp167Tyr	c.499G>T	T1 domain	DEE DEE + EOAE	Rademacher et al. (2020) and Schwarz et al. (2022)
5	p.Phe219Ser	c.656TC	T1 Domain	GGE	Schwarz et al. (2022)
9	p.Val330Met	Undetermined	S3	GGE	Schwarz et al. (2022)
10	p.Ser333Thr	c.998G>C	S3	DEE + Dravet-like syndrome	Seiffert et al. (2023)
11	p.Arg351Lys	c.1052G>A	S4	DEE (CSWS)	Schwarz et al. (2022)
18	p.Phe382Leu	c.1144A>G	S5	DEE	Li et al. (2022)
18	p.Phe388Ser	c.1163T>C	S5	MAE	Schwarz et al. (2022) and Seiffert et al. (2023)
19	p.Phe388Ser	c.1163T>C	S5	DEE	Huo et al. (2023)
20	p.Arg405Gly	c.1213A>G	S5–S6 linker	DEE	Wang et al. (2022)
21	p.Thr437Ala	c.1309A>G	P domain	EOAE	Schwarz et al. (2022)
21	p.Thr437Asn	c.1310C>A	P domain	DEE	Schwarz et al. (2022)
28	p.Leu465Val	Undetermined	S6	Focal epilepsy	Seiffert et al. (2023)
29	p.Val469Leu	c.1405G>T	PVP motif	DEE	Mukherjee et al. (2022)
30	p.Pro470Ser	c.1408C>T	PVP motif	DEE	Li et al. (2022)
31	p.Val471Leu	c.1411G>C	S6	DEE	Vetri et al. (2020), Rydzanicz et al. (2021) and Mukherjee et al. (2022)
32	p.Val473Ala	c.1418T>C	S6	Epilepsy + autism	Mehinovic et al. (2022)
35	p.Asn530His	Undetermined	C terminal	GGE	Seiffert et al. (2023)

CSWS, continuous spikes and waves during sleep; DEE, developmental encephalopathy and epilepsy; EOAE, early-onset absence epilepsy; GGE, genetic generalised epilepsy; MAE, myoclonic astatic epilepsy; PVP motif, proline-valine-proline motif.

**Table 3:** *KCNC3* channel variants and associated clinical presentation.

No. on Figure 1	Protein variant	Coding DNA change	Location in Kv3.3 protein	Clinical presentation	Age of onset (Y)	References
2	p.Asp129Asn	c.385G>A	N terminal	SCA13 + Severe ID	20	Duarri et al. (2015)
7	p.Val340Met	c.1018G>A	S1–S2 extracellular domain	SCA13	46	Tada et al. (2020)
8	p.Arg366His	g.10522G>A*	S2	SCA13	65	Figueroa et al. (2011)
14	p.Arg420His	c.1554G>A c.1259G>A	S4	SCA13	Early, 12–57	Waters et al. (2006), Figueroa et al. (2011), Middlebrooks et al. (2013), Duarri et al. (2015), Pyle et al. (2015), Khare et al. (2017), and Montaut et al. (2017)
15	p.Arg423His	c.1268G>A g.10693G>A*	S4	SCA13 SCA13 + Mild ID	2–4, 17–66	Kim and Sheng (2004), Figueroa et al. (2011), Minassian et al. (2012), Duarri et al. (2015), Khare et al. (2017), Montaut et al. (2017), and Pomarino et al. (2022)
17	p.Phe448Leu	c.1639C>A	S5	SCA13 SCA13 + mild ID + absent seizures	4	Herman-Bert et al. (2000), Waters et al. (2006), and Minassian et al. (2012)
33	p.Val535Met	c.1603G>A	S6	SCA13 + Mild ID	2–3	Duarri et al. (2015)
36	p.Pro583_Pro585del	c.1746_54del	C-terminal	SCA13	Early 30’s	Khare et al. (2017)
37	p.Arg658Gln	c.1973G>A	C-terminal	SCA13	48	Tada et al. (2020)

ID, intellectual disability; SCA13, spinocerebellar ataxia type 13; Y, years; *, genomic DNA change.

### 2.2 Voltage-gated potassium channels in clinical disorders

As highlighted in Tables 1–3, variants in Kv3 channel structure are associated with a wide range of altered functions, manifest as several neurological and psychiatric disorders, of which the major types are described below in more detail. The variants in Kv3 channel function lead to these disease states mechanistically by affecting channel function; for example, changes to the T1 domain can affect the open state stability, important for rapid activation and deactivation (Gunthorpe, 2022).

#### 2.2.1 Epilepsy

Epilepsy is a common complex group of neurological disorders that affect approximately 50 million people globally (Chen, Y.-T. et al., 2023; Chen Z. et al., 2023). Epilepsy is defined as recurrent unprovoked seizures and has many aetiologies and pathophysiologies, including traumatic brain injury, infections, tumours, and stroke (Vezzani et al., 2015). Additionally, gene mutations such as variants in ion channel genes have been associated with epilepsy (Symonds et al., 2017). The diverse aetiology of epilepsy is underpinned by its heterogeneous nature; different types of epilepsies are categorised based on the types of seizures and associated features. Seizures can occur due to the disruption in the excitatory and inhibitory (E-I) balance of brain networks, resulting in an imbalance of excitatory and inhibitory synaptic inputs and increased excitability, as well as hypersynchronisation of neuronal populations (Bertocchi et al., 2023). This synchronicity that precedes a seizure may result from a loss of inhibitory restraint, indicating that seizures can be triggered not only by hyperexcitability but also by a failure of inhibition (Jiruska et al., 2013). In around two-thirds of epileptic cases, the underlying cause is unknown, however, this number is decreasing as the number of monogenic causes discovered has increased (Stenshorne et al., 2022). Various brain areas are associated with seizures including the cerebral cortex, thalamic reticular nucleus, hippocampus, and cerebellum, which are also areas with Kv3-expressing cells (Holmes, 2020; Bernardi et al., 2023).

The voltage-gated K^+^ channel superfamily has been implicated in various forms of epilepsy (Allen et al., 2020). Two main subtypes of epilepsy are associated with Kv3 channel variants: progressive myoclonic epilepsy (PME) and developmental encephalopathy and epilepsy (DEE), as outlined in Tables 1, 2. Despite medical intervention, only around 75% of epileptic patients become seizure-free (McWilliam et al., 2024). However, for a large proportion of epileptic patients, there are currently no treatments available to manage their seizures. Additionally, epilepsy has many psychiatric comorbidities such as ASD and SCZ, suggesting a potential shared pathophysiology.

PME is an umbrella term for a rare collection of autosomal recessive disorders that are characterised by cerebellar ataxia, myoclonus, drug-resistant epilepsy and cognitive decline that gradually worsens over time (Nascimento and Andrade, 2016). Signs of PME tend to emerge in late childhood and early adolescence, typically between the ages of six to fourteen years but can affect any age (Holmes, 2020; Carpenter et al., 2021; Feng et al., 2024). Mutations in *KCNC1* are associated with a specific type of PME, myoclonus epilepsy and ataxia due to potassium channel mutation (MEAK). Kv3.1 undertakes alternative splicing to form two isoforms of the channel, Kv3.1a and Kv3.1b. Kv3.1a expression is greatest during early embryonic development, whereas Kv3.1b is the principal isoform in the adult brain with its expression peaking during adolescence, which overlaps with the onset of PME (Carpenter et al., 2021). A recurrent heterozygous missense *de novo* mutation, c.959G>A (p.Arg320His), has been seen in multiple unrelated patients (Nascimento and Andrade, 2016; Carpenter et al., 2021). This mutation results in the substitution of an arginine residue for a histidine residue in the S4 segment, causing loss of function. This results in mutant channels producing more hyperpolarised potentials and creating barely detectible currents in *Xenopus laevis* oocytes (Muona et al., 2015). When modelled *in vitro* in HEK293 cells (mammalian expression system; Munch et al., 2018) and in neurons (Carpenter et al., 2021), the Kv3.1 mutant channels have a significantly decreased K^+^ current level, and reduced high-frequency firing of the neurons compared to wildtype Kv3.1 channels. Due to the variety of genetic mutations associated with PME, each with mechanistic differences in disease progression, treatment to target the disease mechanism, or slow disease progression, is limited. At present, treating PME is challenging, with current treatment for PME is largely symptom control. Anti-epileptic drugs like valproate are used to manage myoclonic seizures, however, there are no treatments available to manage the cognitive decline seen with the condition (Holmes, 2020). Importantly, pre-clinical research has shown that positive allosteric modulators of Kv3.1 have restored channel function in cell lines expressing the mutated Kv3.1 channel, and phase 1b trials are to continue to investigate these compounds (Autifony, 2024).

Developmental encephalopathy and DEE are a group of severe rare drug-resistant neurological syndromes that are characterised by developmental delay, with or without epilepsy, intellectual disability (ID) with stark abnormal electroencephalogram (EEG) readings (Lin et al., 2022). About 22.2% of children with ID also have some form of epilepsy (McTague et al., 2016). However, in DEE, developmental encephalopathy and epilepsy have evolved independently from each other. Signs of DEE can show within the first few months following birth (Specchio and Curatolo, 2021). Most DEE cases (30–50%) have a sporadic *de novo* cause with over 50 genes associated with DEE (Bertocchi et al., 2023), including genes that encode for various ion channels including potassium channels (*KCNA*, *KCNB* and *KCNQ* subfamilies) (Wang et al., 2022). DEE is implicated with mutations in *KCNC1* and *KCNC2* (Muona et al., 2015; Tables 1, 2). The *KCNC1* variant, p.Ala421Val, is associated with DEE in several patients. This variant alters the function of S6, leading to a loss of whole-cell current and has a dominant negative effect (Cameron et al., 2019; Park et al., 2019). Treatment for DEE is limited to managing epileptic seizures. However, the increasing identification of gene variants associated with DEE and tools such as CRISPR/Cas9 opens the potential for gene therapy and generates preclinical models to further understand mechanisms and subsequent treatment targets (Bertocchi et al., 2023).

#### 2.2.2 Spinocerebellar ataxia

Spinocerebellar ataxia comprises a group of over 40 autosomal dominant neurodegenerative disorders that result in the progressive loss of voluntary movement due to cerebellar atrophy (Shakkottai and Fogel, 2013; Ashizawa et al., 2018) which can impact a patient’s speech, coordination, eye movement and balance. Spinocerebellar ataxia is a heterogeneous disease which is progressive and neurodegenerative and typically affects the cerebellum. It is inherited in an autosomal dominant pattern. Most types of spinocerebellar ataxias (60%) are caused by the abnormal expansion of CAG repeat sequences and ion channel gene mutations that are inherited *de novo*. The numbering of different types of spinocerebellar ataxia is chronologically based on when the associated gene was discovered (Ghanekar et al., 2022). Spinocerebellar type 13 (SCA13) is exclusively due to *de novo* mutations in the *KCNC3* (Kaczmarek and Zhang, 2017; Table 3). Common channel variants include p.Arg420His (R420H) and p.Arg423His (R423H) where an arginine residue is substituted for a histidine in the 420 or 423-position which impacts S4 function (Figueroa et al., 2010; Khare et al., 2017). Co-expression of mutant R420H or R423H in *Xenopus* oocytes results in the suppression of current via a dominant negative mechanism (Irie et al., 2014). R424 mutant mice have a reduced current density, increased basal ${\mathrm{Ca}}^{2+}$ concentration and broadened action potentials, similar to observed outcomes in humans. There are two developmental time points of SCA13 onset, early onset where cerebellar degeneration is seen during early childhood, or adult-onset which presents in middle age and results in slow and progressive loss of motor co-ordination and cognitive decline (Rossi et al., 2014). Late onset is also associated with a disruption in auditory information processing such as locating sounds in space (Middlebrooks et al., 2013). Currently, outcomes are poor with no definitive treatment. There is a multidisciplinary approach to management including neurologists, physiotherapists, occupational therapists and speech and language therapists to help manage the symptoms.

#### 2.2.3 Autism spectrum disorder

ASD can be defined as a heterogeneous collection of neurodevelopmental disorders characterised by impairment in communication, social interaction, and restrictive or repetitive behaviours (NICE, 2021). ASD has an undefined aetiology, believed to be multifactorial, due to complex interactions between genes, environmental factors and epigenetics. It has a prevalence of 1 in 44 children which is increasing (Port et al., 2019; Maenner et al., 2021). ASD patients have depleted numbers of PVI and PV mRNA in post-mortem brains (Hashemi et al., 2017). Furthermore, genetic and environmental risk factor models of ASD also have reduced PVI (Bee et al., 2021). PV knock-out mice show ASD-like behaviours (Wöhr et al., 2015). There is evidence to suggest that voltage-gated K^+^ channels are also implicated in the pathology of ASD. Kv3.1 deficient mice have abnormal social behaviour and hyperactivity, and it has been shown that there are changes to PV in the striatum and prefrontal cortex (Parekh et al., 2018; Bee et al., 2021).

While the majority of ASD aetiology is unclear, the most common single-gene cause of ASD is believed to be due to the silencing of the fragile X messenger ribonucleoprotein 1 (*FMR1*) gene resulting in Fragile X syndrome (5% of ASD cases). Approximately 50–60% of Fragile X patients are also co-diagnosed with ASD (Juarez and Martínez Cerdeño, 2022). *FMR1* is located on the X chromosome (Bhakar et al., 2012). The majority of mutations in *FMR1* are due to an expansion of the CGG repeats in the promoter region. This results in hypermethylation and leads to transcriptional silencing of the *FMR1* gene, reducing the production of Fragile X Messenger Ribonucleoprotein (FMRP), an mRNA-binding protein (Bhakar et al., 2012). FMR1 is strongly expressed in neurons and regulates the expression of various ion channels including binding to mRNA encoding Kv3.1b in brain synaptosomes (Strumbos et al., 2010; El-Hassar et al., 2019). The hallmark characteristics of Fragile X syndrome include ID, extreme hypersensitivity to sensory stimuli (including auditory stimuli), attention deficit and seizures (Strumbos et al., 2010; El-Hassar et al., 2019). Post-mortem human Fragile X brains and *Fmfr1* knockout brains have a significant decrease and lower density of PVI numbers in all brain areas (medial prefrontal cortex (PFC), primary somatosensory cortex, primary motor cortex, superior temporal cortex, and anterior cingulate cortex) (Juarez and Martínez Cerdeño, 2022; Kourdougli et al., 2023). Additionally, hyperactivity of PV neurons has been found in *Fmfr1* knockout mice (Gibson et al., 2008; Nomura et al., 2017; Domanski et al., 2019). There have been efforts to find a successful treatment for Fragile X (Large et al., 2017), however to date, there are no approved medicines.

#### 2.2.4 Schizophrenia

SCZ is a complex disorder without a clear central pathology and is often co-morbid with other conditions such as major depressive disorder, obsessive compulsive disorder, and substance use disorders (Sharma and Reddy, 2019). SCZ exhibits a natural course of progression that often has premorbid impairments and a prodrome, that continues with a relapsing and remitting course (Tandon et al., 2024). SCZ presents with a wide range of symptomology, and due to this, and its various co-morbidities, it is often difficult to diagnose and ascertain an accurate prevalence of the disease. It is a disorder that is one of the top 15 causes of disability worldwide, and can be highly detrimental to individuals (GBD 2016 Disease and Injury Incidence and Prevalence Collaborators, 2017). The main symptoms for those afflicted with SCZ can be classified into three categories: positive symptoms, also referred to as psychotic (hallucinations, delusions, confusion), negative symptoms (alogia, anhedonia, social withdrawal), and cognitive symptoms (working memory impairment, executive function impairment, attention deficit) (Tamminga, 2008).

There is a functional loss of PVI in SCZ and these GABAergic interneurons have a decreased firing rate (Kaar et al., 2019). There are also reduced Kv3.1 containing K^+^ channels in human brain tissue from untreated schizophrenia patients (Yanagi et al., 2014). PVI are usually fast spiking (Nahar et al., 2021), and they fire at a rate capable of entraining gamma band oscillations (30–80 Hz). Therefore, a decreased output from these interneurons leads to gamma band abnormalities, as gamma band activity is governed by PVI (McNally et al., 2013). Indeed, a study found that only cells expressing PV saw an increase in power ratio of local field potential (LFP) in the gamma band activity, which was absent in cells not expressing PV, suggesting that LFP activity in this band frequency is dependent upon this cell type (Smausz et al., 2022). Gamma band abnormalities have been associated with SCZ, with numerous studies linking such abnormalities to the pathology of the disease. An MRI based experiment measuring cortical GABA provides evidence supporting impaired GABAergic neurotransmission in SCZ patients and importantly, correlated this with gamma band activity due to a reduced auditory steady state response in SCZ patients in the gamma band (McNally and McCarley, 2016).

Currently, antipsychotics are mainly used clinically to control the positive symptoms of SCZ, despite cognitive and negative symptoms being predictive of functional outcomes. Pre-clinical research has begun to investigate the Kv3 channels for SCZ to combat the decreased firing of the PVI (Chen, Y.-T. et al., 2023). It is imperative that this research continues so that all three symptom classes can be addressed with new therapies, leading to improved patient outcomes.

## 3 Excitatory—inhibitory imbalance: the underlying mechanism in Kv3 channel-related disorders

**Figure 2:**
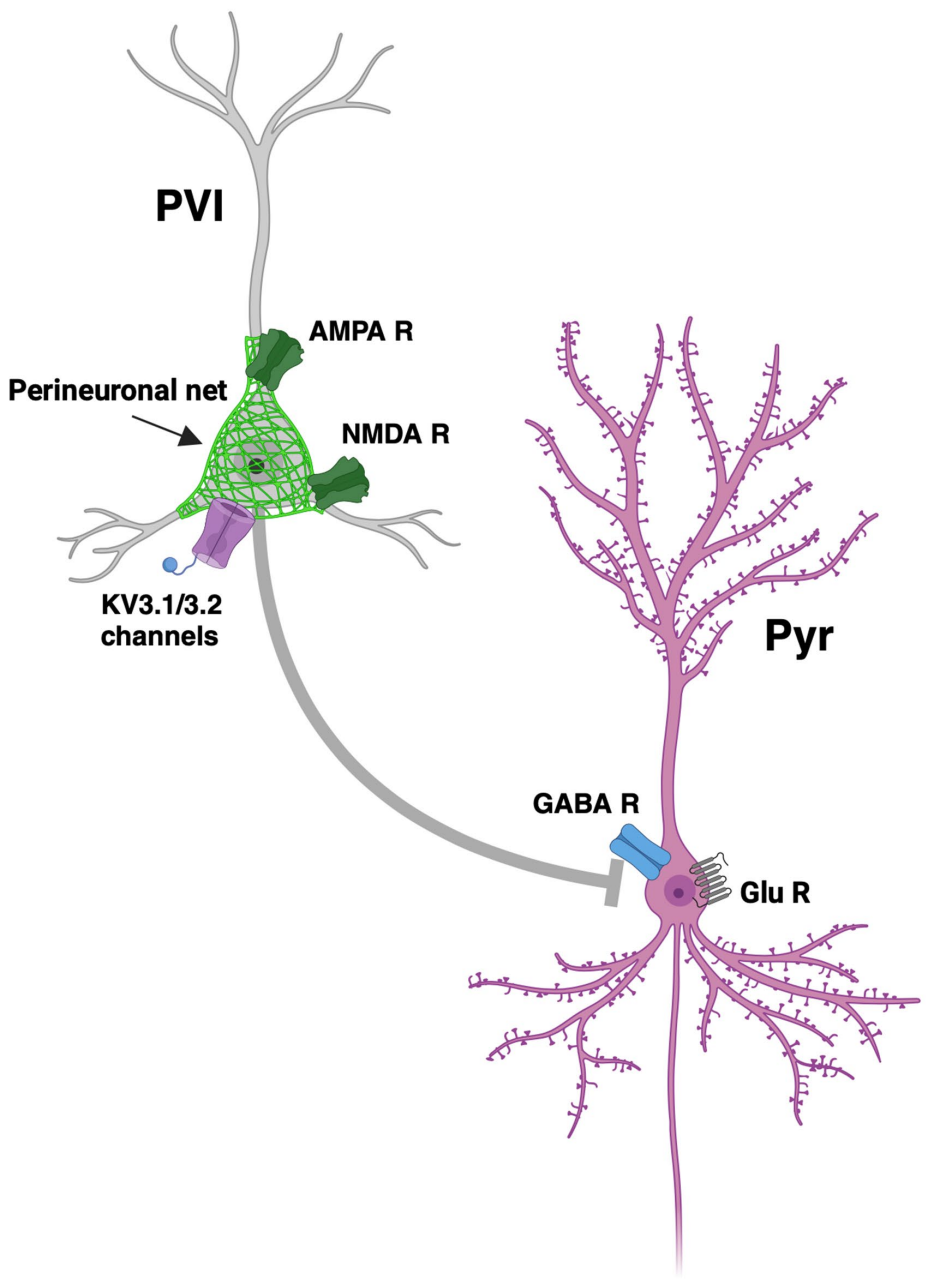
Dysregulation of excitatory-inhibitory imbalance. Functional loss of PVI (parvalbumin expressing interneurons) and hypofunction of NMDA (N-Methyl D-aspartate) receptors on the PVI leads to a reduction in inhibition of Pyr (pyramidal cells) and therefore an increase in glutamate release. Such disruption of excitatory and inhibitory inputs leads to excitatory-inhibitory imbalance. Pictured is a PV interneuron (PVI) covered in a perineuronal net (PNN), represented as a green matrix covering PVI, with an NMDA receptor (NMDA R), and an AMPA receptor (AMPA R), and Kv3.1 and Kv3.2 channels, inhibiting a pyramidal neuron (Pyr) with a γ-aminobutyric acid receptor (GABA R) and glutamate receptors (Glu R) and channels. Figure created using Biorender.com.

It is widely accepted that imbalances in excitatory and inhibitory synaptic currents underlie many psychiatric disorders (Shao et al., 2019; Culotta and Penzes, 2020; Liu et al., 2021). Excitatory activity is mainly glutamatergic driven, while inhibitory activity is GABAergic (Rubenstein and Merzenich, 2003). Inhibitory GABAergic interneurons and excitatory pyramidal cells have often been hypothesised to be at the centre of this imbalance (Figure 2). E-I currents are indeed in a dynamic state, with an imbalance in this, or indeed a change in the E-I ratio, underlying pathological changes relevant to specific symptom domains. Throughout this review we will refer to this imbalance/change in ratio as a balance/imbalance of synaptic E-I inputs in a given system.

High frequency gamma oscillations were reduced in SCZ patients by EEG studies (Lett et al., 2014), and have been proposed to be one of the main driving mechanisms of social and cognitive deficits. Oscillations occur when populations of cells fire in near synchrony. In particular, gamma oscillations (30–80 Hz) are hypothesised to be generated from the pyramidal interneuron network gamma (PING) model, a relationship between pyramidal cells and PV-positive interneurons. Here, the pyramidal cells excite the interneurons using glutamatergic input in a phasic manner, and these currents are synchronised rhythmically by an inhibitory feedback loop (Gonzalez-Burgos and Lewis, 2012; Börgers, 2017). Pyramidal cell inhibition can cause a decrease in gamma oscillation amplitude, which has been linked this to social interaction deficits, implicating gamma oscillations and firing rates as a key locus for social emotion in the PFC (Chen and Hong, 2018; Sato et al., 2023).

E-I balance is underpinned by the relationship between excitatory pyramidal neurons and glutamatergic neurotransmission and the phasic inhibition of these cells by PV-positive interneurons, as shown in Figure 2. Perturbation of this balance of excitatory and inhibitory neurotransmission is central to many psychiatric disorders, underpinned by the altered development of the neurotransmitter systems that contribute to maintaining E-I balance (Pietropaolo and Provenzano, 2022). Dysfunction of this interaction in hippocampal and cortical regions is one of the more consistent alterations in SCZ (Gomes and Grace, 2021). Loss of PVI leads not only to an excitatory overdrive of pyramidal cells but also to dysregulated oscillatory activity across a broad neural network (Lewis et al., 2012). Gamma oscillations in particular depict the functional state and coordinated activity of networks that coordinate cognitive processes, dependent on PVI (Buzsáki and Wang, 2012).

Importantly, E-I imbalance affects hierarchical networks. An example of this are feed forward projections that are associated with hallucinations in SCZ and psychotic disorders. This has been demonstrated by both computational (Jardri and Deneve, 2013), and experimental (Jardri et al., 2017) approaches. Further, the E-I balance is considered to be involved in the default mode network (DMN) and extrinsic cognitive control. These are anti-correlated networks of internally focussed versus outward focussed processes, and the dynamic relationship between them is likely mediated by E-I neurotransmission (Allen et al., 2019). These perturbed network interactions are paramount to the underlying causes of psychiatric symptoms, and are often rooted in childhood trauma and early life insult (Allen et al., 2019).

### 3.1 Parvalbumin-expressing interneurons

Both human and animal studies have shown that E-I imbalance is perpetuated by a reduction of PVI, which is linked to oscillatory activity differences (Lodge et al., 2009). Reduced levels and expression of PV explains a decrease in PVI output and therefore a loss of the fast-spiking phenotype of these cells. Without the fast-spiking ability of the PVI they are not able to entrain gamma rhythms. Thus, PVI, and their fast-spiking ability, are a potential target for drug development. Cardin et al., 2009, demonstrated that the PV expressing cells (i.e., fast-spiking cells) are responsible for gamma rhythm generation (Cardin et al., 2009). Here, they drove a cortical network of fast spiking cells in PV-Cre mice (PVI) and regular spiking cells in αCamKII-Cre mice (including pyramidal neurons) at a range of frequencies between 8–200 Hz with 1 ms light pulses (Cardin et al., 2009). Only at the gamma range (40 Hz) did they see an increase in local field potential power ratio from the fast spiking PVI, demonstrating that gamma rhythms are dependent on PV (Cardin et al., 2009).

Furthermore, studies have shown a reduction in GAD67 mRNA, the gene responsible for the synthesis of GABA, in post-mortem human brains of patients with SCZ and mood disorders (Thompson et al., 2009). In the maternal immune activation model (mIA) model, there is a reduction in *Arx* gene expression, which is critical to PVI development (Nakamura et al., 2019). We also note that there are morphological and functional changes to the PVI in different types of epilepsy (Ye and Kaszuba, 2017), and a reduced number of PVI in sections of post-mortem ASD brain (Hashemi et al., 2017).

Taken together, the accumulating evidence highlights that PVI should be considered as a main target for the modulation of the E-I imbalance, as depicted in Figure 2. Therefore, in further drug development, research on voltage-dependent potassium channels has gained interest, as these channels are expressed on the PVI. Two voltage-gated potassium channels, Kv3.1 and 3.2 are co-localised with PVI (McDonald and Mascagni, 2006), as shown in Figure 2. Potentiating these channels through pharmacological intervention could hold promise for the treatment of cognitive and social deficits found in neurological disease.

## 4 Kv3 channels as a potential therapeutic drug target

Neuronal cell characteristics are shaped by the potassium channels they express. Neurons in the auditory brain stem, fast-spiking GABAergic PVI, and the Purkinje cells of the cerebellum all express potassium channels from the Kv3 family (Kaczmarek and Zhang, 2017). These channels have particularly high activation potentials and rapid kinetics, activating and deactivating to release neurotransmitters from the interneurons at rates of up to 1,000 Hz (Kaczmarek and Zhang, 2017; Chen et al., 2023a). This is achieved by detecting changes in the membrane potential and repolarising the cell. Cryo-EM structural characterisation found a binding site for positive allosteric modulators, providing the potential for a Kv3 channel modulator to be developed (Botte et al., 2022).

One of the challenges in developing a Kv3 channel modulator is conferring specificity of action to the desired channel. Lack of structural high resolution for Kv3 channels has previously hindered development of a targeted therapeutic, but recent cryo-EM studies have now characterised the Kv3.1 binding sites (Botte et al., 2022; Liang et al., 2024). Botte et al. (2022) identified a difference between the Kv1.2 and Kv3.1 structures, which are very similar, at the intracellular T1 domain, and this distinction facilitated development of a specific Kv3.1 modulator. Further, Boddum et al. (2017) used *Xenopus laevis* oocyte electrophysiology specificity studies to confirm that a widely used positive allosteric modulator specifically modulated Kv3.1 and partially Kv3.2. This compound has been developed further to be highly specific to Kv3.1 and Kv3.2, and is now widely used in the field (Liang et al., 2024).

Kv3 channel modulators work by lowering the voltage activation of the fast spiking GABAergic PVI closer to that of a normal action potential in an *ex vivo* mouse model (Brown et al., 2016). They enable the fast-spiking phenotype of the cells to be rescued, demonstrated in rat hippocampal studies, where the firing rate was increased in GABAergic interneurons (Boddum et al., 2017). This makes this class of drugs a novel avenue for restoring the E-I balance. Andrade-Talavera et al. (2020) used the neurotoxic amyloid beta protein 42 to reduce cognitive-relevant gamma oscillatory activity in fast spiking interneurons. When the Kv3 modulator was applied it resulted in faster activation kinetics and an increased firing rate, correlated with gamma normality. Autifony Therapeutics^1^ have been developing a Kv3.1b and Kv3.2 channel modulator compound, currently known as AUT00206, that is investigated in a number of disorders with underlying cognitive and social deficits such as Fragile X, epilepsy, hearing disorders, and SCZ, which may all have the common underlying mechanism of E-I imbalance.

Recent studies have shown that ketamine-challenged patients (an NMDA receptor antagonist) had an increased blood oxygen level dependent (BOLD) signal (an index of neuronal activity), which was subsequently reduced with Kv3 channel positive allosteric modulator (PAM) treatment (Deakin et al., 2019). Moreover, it normalised gamma oscillations in schizophrenic patients, further suggesting that this may be the underlying cause of cognitive impairment (Ben-Mabrouk et al., 2014). Kv3 modulators have been used to restore certain cognitive phenotypes such as motor function in an epilepsy model (Feng et al., 2024), and reversal learning deficits in a SCZ model (Leger et al., 2014a). A summary of *in vitro*, *in vivo*, and human studies where Kv3-modulating drugs have been used is shown below in Table 4.

**Table 4:** A list of studies in which Kv3 channel modulators have been used, highlighting the disease model and the main outcome measured.

Type of study	Disease type	Main outcome	References
*In vitro*, mouse	AD	Kv3.1/3.2 on FSN modulation results in faster activation kinetics	Andrade-Talavera et al. (2020)
*In vitro*, mouse	Hearing disorders	Kv3.1 modulators shift activation potential	Brown et al. (2016)
*In vitro*, mouse	Kv3 channels blocked	Kv3 modulator rescues fast-spiking phenotype	Rosato-Siri et al. (2015)
*In vivo*, mouse	Fragile X	Kv3 modulator reversed deficit in NOR, hyperlocomotion, and freezing in fear conditioning	Large et al. (2017)
*In vivo*, mouse	Fragile X	Kv3 modulator can retrieve circuit dynamics and tactile defensiveness	Kourdougli et al. (2023)
*In vivo*, mouse	Fragile X	Kv3 modulator decreases the firing rate and improves the auditory brainstem response	El-Hassar et al. (2019)
*In vivo*, mouse	Myoclonus epilepsy	Kv3 modulator improves motor function and seizure susceptibility	Feng et al. (2024)
*In vivo*, rat	Age-related hearing loss	Kv3 modulators improved gap detection	Rybalko et al. (2021)
*In vivo*, mouse	Hearing disorders	Kv3 modulator normalises abnormal pathology in inferior colliculus	Anderson et al. (2018)
*In vivo*, hamsters	Tinnitus	Kv3 modulators suppress tinnitus-related hyperactivity	Glait et al. (2018)
*In vivo*, *in silico*, mouse	Auditory nerve damage	Kv3 modulators reduced action potential timing variability and improved temporal coding precision	Chambers et al. (2017)
*In vivo*, rat	Schizophrenia	Kv3 modulator reverses cognitive and neurobiological dysfunction	Leger et al. (2015)
*In vivo*, mouse	Bipolar disorder	Kv3.1/3.2 modulator reverses hyperactivity	Parekh et al. (2018)
*In vivo*, rat	Schizophrenia	Kv3.1 modulator attenuates reversal learning deficit	Leger et al. (2014b)
*In vivo*, rat	Schizophrenia	Kv3.1 modulator attenuates reversal learning deficit	Harte et al. (2014)
*In vitro*, mammalian neocortex	Schizophrenia	Increases power and area power of gamma oscillations	Ben-Mabrouk et al. (2014)
Human	Schizophrenia	Reduction in schizophrenia symptoms	Kaar et al. (2022)
*In vivo*, rat	Fear discrimination	Regulates fear discrimination	Stubbendorff et al. (2023)
Human	Ketamine-induced BOLD signalling	Reduces BOLD signal in response to ketamine	Deakin et al. (2019)

AD, Alzheimer’s disease; BOLD, blood oxygen level dependent; FSN, fast spiking neuron; NOR, novel object recognition.

## 5 Discussion

Voltage-gated potassium channels of the Kv3 subfamily are central to synaptic transmission due to their location on fast spiking neurons. These channels therefore are likely to contribute significantly to neurological and psychiatric disorders, many of which are characterised by cognitive deficits. Currently, treatment for key symptoms of many of these disorders is limited, with many therapeutics focused on symptomatic relief as opposed to directly targeting the underlying pathological mechanism. Therefore, there is an urgent need to find novel treatments for disorders with a strong cognitive component. Recent data also draw attention to the possibility that these channels can be utilised as a therapeutic avenue for treating neurological and psychiatric disorders, which would address an unmet clinical need.

Together, the collective evidence presented in this review highlights the central role of voltage-gated Kv3 channels in maintaining E-I balance. Hence, there is the exciting possibility of targeting Kv3 channel function as a new locus for novel treatment, whilst also advancing our understanding of the mechanisms by which such deficits give rise to E-I imbalance that underpin a variety of neurodevelopmental, neurological, and psychiatric disorders.

## Footnotes

^1^https://autifony.com/science/
